# Genome-Wide Identification and Functional Characterization of the Trans-Isopentenyl Diphosphate Synthases Gene Family in *Cinnamomum camphora*

**DOI:** 10.3389/fpls.2021.708697

**Published:** 2021-09-13

**Authors:** Zerui Yang, Chunzhu Xie, Ting Zhan, Linhuan Li, Shanshan Liu, Yuying Huang, Wenli An, Xiasheng Zheng, Song Huang

**Affiliations:** ^1^School of Pharmaceutical Sciences, Guangzhou University of Chinese Medicine, Guangzhou, China; ^2^National Engineering Research Center for Healthcare Devices, Institute of Medicine and Health, Guangdong Academy of Sciences, Guangzhou, China,; ^3^National Engineering Research Center for Modernization of Traditional Chinese Medicine, Guangzhou University of Chinese Medicine, Guangzhou, China

**Keywords:** genome-wide identification, *Cinnamomum camphora*, trans-isopentenyl diphosphate synthases, functional characterization, gene family

## Abstract

Trans-isopentenyl diphosphate synthases (TIDSs) genes are known to be important determinants for terpene diversity and the accumulation of terpenoids. The essential oil of *Cinnamomum camphora*, which is rich in monoterpenes, sesquiterpenes, and other aromatic compounds, has a wide range of pharmacological activities and has therefore attracted considerable interest. However, the *TIDS* gene family, and its relationship to the camphor tree (*C. camphora* L. Presl.), has not yet been characterized. In this study, we identified 10 *TIDS* genes in the genome of the *C. camphora* borneol chemotype that were unevenly distributed on chromosomes. Synteny analysis revealed that the *TIDS* gene family in this species likely expanded through segmental duplication events. Furthermore, cis-element analyses demonstrated that *C. camphora* TIDS (CcTIDS) genes can respond to multiple abiotic stresses. Finally, functional characterization of eight putative short-chain TIDS proteins revealed that CcTIDS3 and CcTIDS9 exhibit farnesyl diphosphate synthase (FPPS) activity, while CcTIDS1 and CcTIDS2 encode geranylgeranyl diphosphate synthases (GGPPS). Although, CcTIDS8 and CcTIDS10 were found to be catalytically inactive alone, they were able to bind to each other to form a heterodimeric functional geranyl diphosphate synthase (GPPS) *in vitro*, and this interaction was confirmed using a yeast two-hybrid assay. Furthermore, transcriptome analysis revealed that the *CcTIDS3*, *CcTIDS8*, *CcTIDS9*, and *CcTIDS10* genes were found to be more active in *C. camphora* roots as compared to stems and leaves, which were verified by quantitative real-time PCR (qRT-PCR). These novel results provide a foundation for further exploration of the role of the *TIDS* gene family in camphor trees, and also provide a potential mechanism by which the production of camphor tree essential oil could be increased for pharmacological purposes through metabolic engineering.

## Introduction

*Cinnamomum camphora* is a subtropical evergreen tree species that has been widely cultivated in southern China for over 1,500years (Chen et al., 2020b). Studies have shown that essential oil extracted from the leaves of the camphor tree, which is rich in monoterpenes, sesquiterpenes, and other aromatic compounds, has a wide range of pharmacological activities, including antibacterial, antioxidant, and insecticidal properties (Yu et al., 2019).

*Cinnamomum camphora* subspecies can be grouped into five chemotypes according to the dominant component in their essential oils, which were extracted from their leaves: linalool, D-borneol, camphor, cineole, or nerolidol (Guo et al., 2017; Chen et al., 2018). These terpenoids have important industrial and pharmaceutical applications. For example, D-borneol is a well-established traditional Chinese medicine that is used to treat cardiovascular diseases, including stroke, coronary heart disease, and angina pectoris (Yang et al., 2020). D-Borneol has been documented in various versions of the Chinese Pharmacopoeia (Huang et al., 2016; Liang et al., 2018; Ren et al., 2018; Chai et al., 2019; Chen et al., 2019; Yang et al., 2020), and it is in high demand as a key ingredient in many traditional Chinese herbal formulas. This compound can also be used to relieve pain resulting from wounds, injuries, burns, and cuts (Yang et al., 2020). However, despite the high medicinal value of volatile terpenoids, limited plant resources, and low extraction efficiency limit the amounts of the essential oils from *C. camphora* that can be obtained for research or practical applications (Ma et al., 2021).

An increasing body of research has demonstrated that metabolic engineering consists of optimizing the genetic and regulatory mechanisms that govern cellular processes, and this is an effective method that can be used to increase the production of active natural products in microorganisms and plants (Choi et al., 2019; Li and Mutanda, 2019). Identifying the genes that govern production and accumulation of the essential oil in *C. camphora* could therefore enable the implementation of this approach to increase the essential oil yield, which would assist in meeting high demand.

Terpenoids are the largest category of plant specialized metabolites. More than 55,000 of these compounds have thus far been described, and are grouped into hemi- (C5), mono- (C10), sesqui- (C15), di- (C20), sester- (C25), tri- (C30), tetra- (C40), and poly- (C50) terpenoids according to the number of carbon atoms they contain (Dudareva et al., 2004; Pichersky et al., 2006; Ueoka et al., 2020). Despite diverse functions and structures, all terpenoids contain two universal C5 units: isopentenyl diphosphate (IPP) and its allylic isomer dimethylallyl diphosphate (DMAPP). The mevalonic acid (MVA) pathway gives rise to IPP, and *via* enzymatic isomerization, to DMAPP, whereas the methylerythritol phosphate (MEP) pathway directly produces both IPP and DMAPP. IPP and DMAPP can be condensed (head to tail) by prenyltransferase (PTS) or isoprenyl diphosphate synthase (IDS), resulting in a series of prenyl diphosphates with various chain lengths. These linear precursors are then catalyzed by terpene synthase (TPS) and other modifying enzymes to form a variety of different terpenoids (Tholl and Lee, 2011; Athanasakoglou and Grypioti, 2019; Johnson et al., 2019; Johnson and Bhat, 2019; Adal and Mahmoud, 2020; Hivert et al., 2020; Miller and Bhat, 2020).

Isoprenyl diphosphate synthase is located at the branch point of the terpenoid biosynthetic pathway, and plays a vital role in the formation of diverse terpenoid structures (Jia and Chen, 2016). Differences in terpenoid synthase gene expression and the supply of precursors determine the terpenoid composition produced by plants (Dudareva et al., 2000; Johnson et al., 2019; Johnson and Bhat, 2019; Adal and Mahmoud, 2020; Miller and Bhat, 2020). Both trans- and cis- isomers of the products of IDS exist (Liang et al., 2002). Trans-IDS (TIDS) enzymes synthesize isoprenyl diphosphates (Barja and Rodríguez-Concepción, 2020) and are classified as short-chain (SC-TIDS, C10–20), medium-chain (MC-TIDS, C25–35), or long-chain (LC-TIDS, C40–50), depending on the length of the isoprenyl diphosphates that they produce (Wang et al., 2019). Cis-IDSs (CIDSs) were initially predicted to synthesize long-chain isoprenyl diphosphates (>C50) for dolichol and polyprenol production (Jia and Chen, 2016). Although, TIDSs and CIDSs have similarities in substrate preference and reaction products, they utilize different catalytic mechanisms and may be readily distinguished from one another by their primary amino acid (AA) sequences (Akhtar et al., 2013).

Short-chain-trans-isopentenyl diphosphate synthase includes homodimeric or heterodimeric geranyl diphosphate synthase (GPPS), farnesyl diphosphate synthase (FPPS), and geranylgeranyl diphosphate synthase (GGPPS; Jia and Chen, 2016), and their products provide the precursors for monoterpenes, sesquiterpenes, and diterpenes, respectively. Geranylfarnesyl diphosphate synthase (GFPPS) and polyprenyl diphosphate synthase (PPPS) are MC-TIDSs, whereas solanesyl diphosphate synthase (SPPS) is classified as an LC-TIDS (Vandermoten et al., 2009; Nagel et al., 2015; Wang et al., 2016; Kopcsayová and Vranová, 2019).

Phylogenetic analysis has revealed that plant *TIDS* genes can be documented into five subfamilies according to sequence identity: *TIDS-a*, *-b*, *-c*, and *-e* including the genes encoding FPPS, SPPS, PPPS, and small subunits (SSUs) of GPPS, respectively (Jia and Chen, 2016). The *TIDS-d* subfamily is more complex, and it includes genes encoding GGPPS, GFPPS, and PPPS that share a high sequence identity of at least 40%, with some shared identities of 55% or greater (Jia and Chen, 2016; Cui et al., 2019). Identification of entire *TIDS* gene families in plant species is required to determine their functions and to understand their combined effect on the specific profile of terpenoids produced.

Thus far, this level of *TIDS* gene family characterization has only been achieved in the model plant *Arabidopsis thaliana* (Kopcsayová and Vranová, 2019), in which 16 *TIDS* genes were identified, and in the tomato plant *Solanum lycopersicum* (Zhou and Pichersky, 2020), in which 10 were characterized. Studies characterizing these *TIDS* genes have revealed that while most of the synthase types they encode are homodimeric, GPPS may exist as either a homodimer or a heterodimer containing a small subunit (SSU) and a large subunit (LSU), or both (Rai et al., 2013; Chen et al., 2015; Adal and Mahmoud, 2020). The SSU can be further separated into two types (I and II), which are generally inactive alone. The LSU may be either inactive alone or possess GGPPS activity, while its heterodimer is an active GPPS (Burke et al., 2004; Rai et al., 2013).

Given that isoprenyl diphosphate biosynthesis is a crucial determinant for the formation of downstream terpenoid type and their yield, in-depth studies on TIDSs in the camphor tree would be instrumental in optimizing the production of these medicinally valuable products. Here, for the first time, we conducted a genome-wide analysis in the borneol chemotype of *C. camphora* to identify and characterize TIDS genes and the proteins they encode.

## Materials and Methods

### Plant Materials

Borneol chemotype *C. camphora* plants were purchased from Ji’an Yu Feng Natural Species Co., Ltd. (Ji’an, China), and grown in an artificial climate box (Shanghai Yiheng Instrument Co., Ltd., Shanghai, China) at 25°C with a 12h light and then 12h dark photoperiod. We verified the origin of these plants through DNA barcodes.

### Genome-Wide Identification of TIDS Genes in *C. camphora*

We downloaded TIDS protein sequences of *A. thaliana* and *S. lycopersicum* from The Arabidopsis Information Resource (TAIR)^1^ and the Sol Genomics Network (Wijfjes and Smit, 2019).^2^ We then used the Protein Basic Logical Alignment Search Tool (BLASTP, United States National Library of Medicine)^3^ to compare these sequences with the *C. camphora* database (unpublished), using previously established thresholds for *e*-value (≤1e^−5^) and identity (Cui et al., 2019). Any genes identified were then used as queries for a second round of BLASTP searches to ensure no putative *CcTIDS* genes were missed. We also conducted a Hidden Markov Model (HMM) search for sequence homologs using the HMMER 3.0 program and the polyprenyl synthase domain PF00348 as a bait, and previously established *e*-value and identity thresholds (Wong et al., 2015; Cui et al., 2019).

The BLASTP and HMM search results were then integrated to identify candidate TIDS genes. Their sequences were then submitted to the online Pfam database and NCBI conserved domains database (CDD) in order to verify the presence of the polyprenyl synthase domain (Zhu et al., 2020). The physicochemical parameters of each CcTIDS, including molecular weight and isoelectric point, were calculated using the ExPASy online tool (Zhu et al., 2020).^4^ The MEME online tool^5^ was used to discover conserved domains in the amino acid sequence of each CcTIDS (Zhu et al., 2020). Finally, we used the chloroP (Emanuelsson et al., 1999),^6^ TargetP (Emanuelsson et al., 2000),^7^ Wolfpsort (Horton et al., 2007),^8^ and Plant-mPloc (Chou and Shen, 2010)^9^ tools to predict the subcellular localization of each CcTIDS.

### Phylogenetic Relationship, Exon-Intron Structure, Chromosomal Localization, and Cis-Acting Element Analysis

A maximum likelihood (ML) evolutionary tree was constructed using the identified *C. camphora* TIDS amino acid sequences and those of 10 other plant species (*A. thaliana*: Arabidopsis Genome Initiative, 2000; *Oryza sativa*: Yu et al., 2002; *Physcomitrella patens*: Rensing et al., 2008; *Selaginella moellendorffii*: Banks et al., 2011; *S. lycopersicum*: Tomato Genome Consortium, 2012; *Amborella trichopoda*: Amborella Genome Project, 2013; *Picea abies*: Nystedt et al., 2013; *Zea mays*: Jiao et al., 2017; and *Cinnamomum micranthum*: Chaw et al., 2019) and the Molecular Evolutionary Genetics Analysis (MEGA) tool (version 7.0; Kumar et al., 2016) with the best models of JTT+G. Bootstrap value, which indicates the reliability of each branch node, was set at 1,000 replicates. The resulting tree was visualized using EvolView v3 (Subramanian et al., 2019).

Exons and introns for each TIDS were identified using the gene transfer format (GTF) file from the *C. camphora* genome, which contains information regarding gene structure, and visualized using TBtools (Chen et al., 2020a). Finally, in order to identify cis-regulatory elements, TBtools was used to extract the 2,000bp upstream sequence for each TIDS gene identified in the *C. camphora* genome (Chen et al., 2020a). We then compared these sequences with the PlantCARE database of plant cis-acting regulatory elements (Lescot et al., 2002). Circos graphs showing the chromosomal localization and results of the synteny analysis of the CcTIDS sequences were drawn using TBtools (Chen et al., 2020a).

### RNA Extraction, cDNA Synthesis, and Gene Cloning

Total RNA was extracted from the root, stem, and leaf of *C. camphora* using the Plant Pure Plant RNA Kit (Aidlab, Beijing, China). cDNA was synthesized from 1μg of high-quality total RNA (OD 260/280=1.8–2.2, OD 260/230≥2.0, RIN≥6.5, and 28S:18S≥1.0) using the TransScript One-Step gDNA Removal and cDNA Synthesis SuperMix kit (TransGen Biotech, Beijing, China) according to the manufacturer’s instructions (Su et al., 2019).

The primers use to amplify eight putative SC-TIDSs were designed using the Primer Premier 5 program (Supplementary Table S4). Amplification by PCR was conducted using the 2×TransStart FastPfu PCR SuperMix (TransGen Biotech, Beijing, China) and cDNA as the template. Purified PCR products were then cloned into the pEASY-blunt vector using previously described methods (Su et al., 2019).

### Quantitative Real-Time PCR Analysis

Quantitative real-time PCR (qRT-PCR) was performed using the TransStar Tip Green qPCR SuperMix (TransGen Biotech, Beijing, China) and the CFX96 Touch Deep Well platform (Bio-Rad, United States; Yang et al., 2020). We used a previously described reaction system composition and qRT-PCR procedure (Yang et al., 2020). Primers are listed in Supplementary Table S4.

### Subcellular Localization of CcTIDS Proteins

Full-length putative SC-TIDS gene sequences without stop codons were each fused with enhanced green fluorescent protein (EGFP) and ligated into the pAN580 vector (using the primers described in Supplementary Table S4). The recombinant vectors were then transformed into *Arabidopsis* protoplasts using polyethylene glycol (PEG; Zhou and Pichersky, 2020). Protoplast isolation and recombinant vector transformation were performed with protocols described in previous studies (Yoo et al., 2007; Wang et al., 2018). EGFP fluorescent signals were observed using a Zeiss laser scanning microscope (LSM) 800 (Zeiss, Germany) as previously described (Beck et al., 2013; Su et al., 2019).

### Recombinant Expression and Enzymatic Assays

Truncated or full length versions of the eight putative SC-TIDSs were ligated into the pET32a [polyhistidine (6x His) tag, Wego, Guanghzou, China] or pMAL-C5X (MBP tag, Wego, Guanghzou, China) expression vectors using the pEASY-Basic Seamless Cloning and Assembly Kit (TransGen Biotech) following the manufacturer’s instructions (Su et al., 2019), using the primers listed in Supplementary Table S4. Constructs were verified using Sanger sequencing. Upon sequence confirmation, recombinant plasmids were transferred into the expression strain *Escherichia coli* Rossetta (DE3; Huayueyang, Beijing, China). Heterologous protein expression in this strain and the purification of the fusion protein were conducted according to previously established methods (Su et al., 2019).

In order to establish the function of each SC-TIDS, a 200-μl *in vitro* enzymatic activity reaction containing 25mmol/L MOPSO buffer with pH of 7.0, 10mmol/L magnesium chloride, 10% glycerin, 20–50μg protein, and substrates (150μmol/L IPP+40μmol/L DMAPP or GPP or FPP, Sigma-Aldrich, United States), was conducted. Each mixture was first incubated at 30°C for 6h, and then, 200μl of 200mmol/L Tris–HCl (pH 9.5), containing 2units of bovine intestine alkaline phosphatase (18unitsmg^−1^; Sigma-Aldrich) and 2units of potato apyrase (25.2unitsmg^−1^; Sigma-Aldrich) were added (Rai et al., 2013). An overnight hydrolysis reaction was carried out at 30°C. Ethyl acetate was then used to extract the enzymatic reaction buffer in 2×400μl extractions (Su et al., 2019). The ethyl acetate extracts were concentrated to 100μl under N_2_, and these concentrated extracts were then used for gas chromatography–mass spectrometry (GC–MS) analysis using previously described methods (Athanasakoglou and Grypioti, 2019; Yang et al., 2020). The GPPS large and small subunits from *Catharanthus roseus* were also analyzed as a positive control (Rai et al., 2013).

### Yeast 2-Hybrid Assay

The interactions between the CcGPPS small and large subunits were verified by the following experiments: the truncated versions of these subunits were amplified from the cDNA of *C. camphora*, followed by fusing to the activation domain of the pGADT7 vector or the binding domain of the pGBKT7 vector. Recombinant vectors were co-transformed into *Saccharomyces cerevisiae* AH109 yeast and sequentially cultivated on the synthetic dropout (SD) medium SD/-Trp/-Leu. The interaction between the two proteins was tested on SD/-Trp/-Leu/-His/-Ade medium supplemented with 150mM 3-amino-1,24-triazole (3-AT).

## Results

### Genome-Wide Identification of TIDS Genes in *C. camphora*

**Figure 1:**
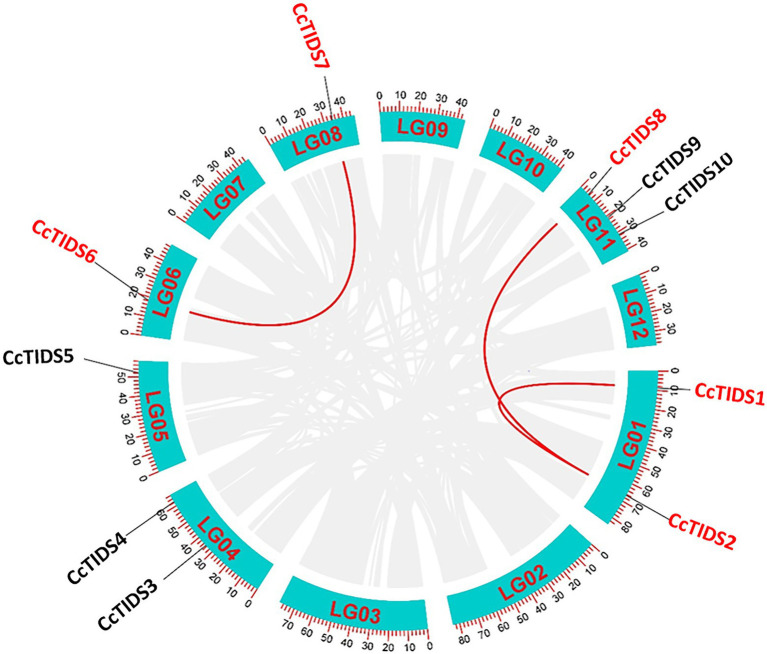
Circos graph showing the chromosomal locations and duplicated gene pairs of trans-isopentenyl diphosphate synthases (*CcTIDS*) genes in the *C. camphora* genome. The blue segments represent the 12 chromosomes present in this species. Duplicated genes are indicated as red lines between each gene pair.

Based on the HMM scan and BLASTP search results, 10 full-length, protein-coding TIDS-like gene sequences were identified in *C. camphora*, which we numbered *CcPTPS1–10* according to their locations on the chromosomes. These genes included eight putative SC-TIDS (two FPPSs, *CcTIDS3* and *CcTIDS9*; two GPPSs, *CcTIDS4* and *CcTIDS5*; three GGPPSs, *CcTIDS1*, *CcTIDS2*, and *CcTIDS8*; and one GPPS small subunit, *CcTIDS10*) and two putative LC-TIDS (SPPS, and *CcTIDS6* and *CcTIDS7*), which were distributed along six of the 12 chromosomes (Figure 1). In addition, genome synteny analysis showed that three segmental duplications and no tandem duplication events likely occurred, suggesting that segmental duplication might be one of the reasons for TIDS gene family expansion (Figure 1).

The proteins encoded by the *CcTIDS* genes were found to have a minimum size of 298 AA and a maximum size of 428 AA. All of the CcTIDS proteins had an isoelectric point (pI) <7, indicating that these proteins are rich in acidic AAs. Specific information for all CcTIDS proteins is shown in Table 1.

**Table 1:** Nomenclature and physicochemical characteristics of TIDS genes identified in *C. camphora*.

Gene ID	Rename	Chromosomal position	Putative function	Number of amino acids/AA	Theoretical pI	Molecular weight/KD	*In silico* subcellular localization prediction				Conserved motif
							chloroP	TargetP	Wolfpsort	Plant-mPloc	
evm.model.LG01.0506	CcTIDS1	LG01	GGPPS	383	5.98	41.34	Chloroplast	Chloroplast	Chloroplast	Chloroplast	FARM/SARM/CXXXC
evm.model.LG01.2465	CcTIDS2	LG01	GGPPS	387	6.33	41.7	Chloroplast	Chloroplast	Chloroplast	Chloroplast	FARM/SARM/CXXXC
evm.model.LG04.0985	CcTIDS3	LG04	FPPS	350	5.25	40.41	/	/	cytoplasm	Mitochondrion.	FARM/SARM
evm.model.LG04.2634	CcTIDS4	LG04	GPPS	421	5.67	46.45	/	Mitochondrion	Chloroplast	Chloroplast	FARM/SARM
evm.model.LG05.1976	CcTIDS5	LG05	GPPS	428	6.09	47.04	/	Mitochondrion	Mitochondrion	Chloroplast	FARM/SARM
evm.model.LG06.0936	CcTIDS6	LG06	SPPS	415	6.42	45.3	/	/	Chloroplast	Chloroplast	FARM/SARM
evm.model.LG08.1085	CcTIDS7	LG08	SPPS	401	5.50	43.68	/	Mitochondrion	Chloroplast	Chloroplast	FARM/SARM
evm.model.LG11.0380	CcTIDS8	LG11	GGPPS	383	5.78	41.39	Chloroplast	Chloroplast	Chloroplast	Chloroplast	FARM/SARM/CXXXC
evm.model.LG11.1115	CcTIDS9	LG11	FPPS	364	5.18	41.45	/	Chloroplast	Chloroplast	Mitochondrion	FARM/SARM
evm.model.LG11.1288	CcTIDS10	LG11	GPPS.SSU	298	5.24	32.13	Chloroplast	Chloroplast	Chloroplast	Chloroplast	CXXXC\CXXXC

### Phylogenetic Relationships Within the *C. camphora* TIDS Gene Family

A total of 100 *TIDS*-like genes were identified in 10 other plant species (Supplementary Tables S1 and S2). It can be concluded from the number of TIDS genes contained in each species that as the complexity of the species increases, the greater amount TIDS it contains (Coman et al., 2014). In addition, fewer *CcTIDS* genes were found in *C. camphora* than those in *A. thaliana*, *Populus trichocarpa*, *O. sativa*, and *Z. mays*, but equal to those in *S. lycopersicum* and the stout camphor tree *C. micranthum*.

**Figure 2:**
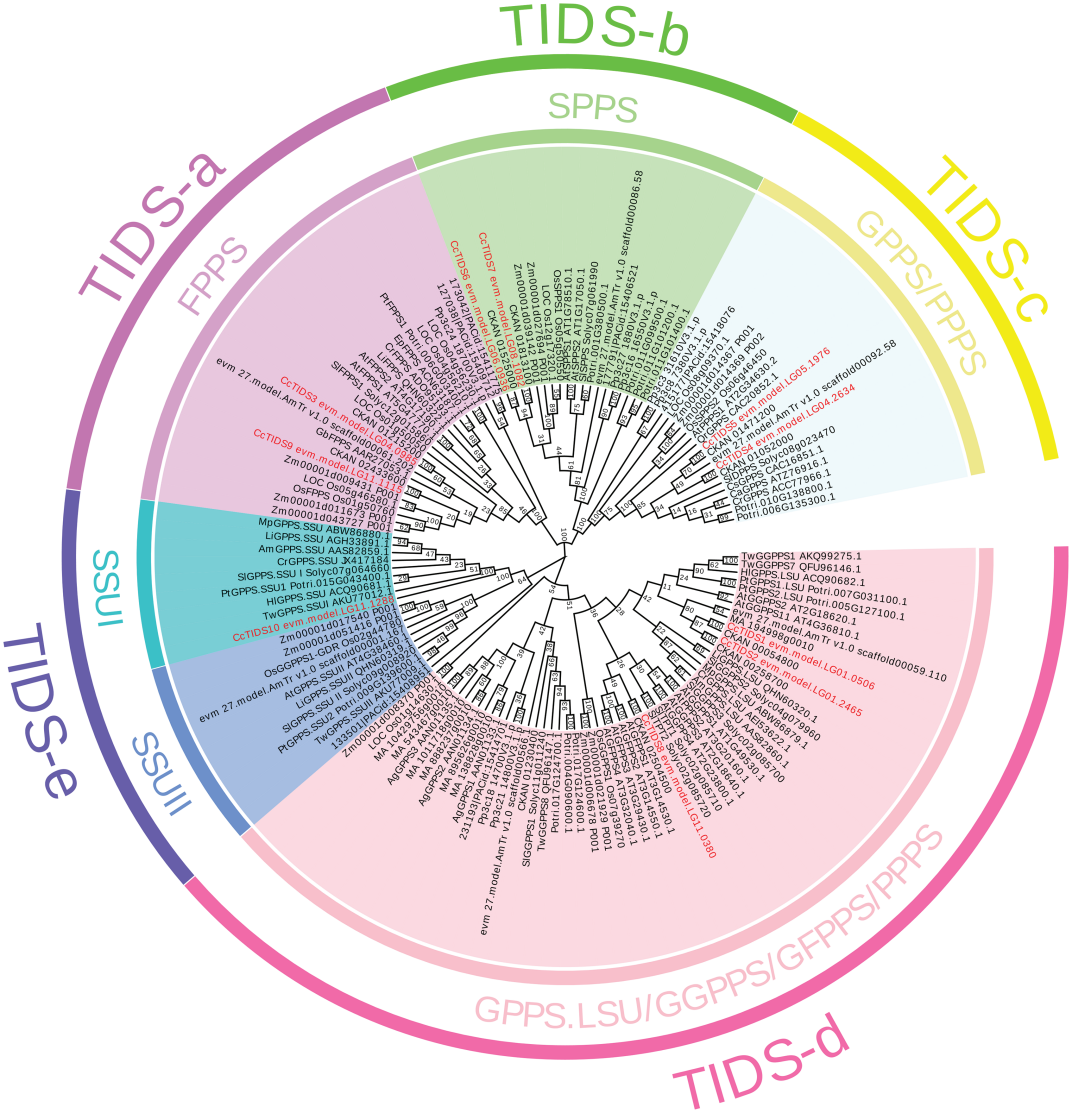
A phylogenetic tree constructed using putative or characterized *TIDS* genes from 11 sequenced land plant genomes and a neighbor joining approach. GPPS, geranyl diphosphate synthase; FPPS, farnesyl diphosphate synthase; GGPPS, geranylgeranyl diphosphate synthase; LSU, large subunit; SSUI/II, small subunit I/II; GFPPS, geranylfarnesyl diphosphate synthase; PPPS, polyprenyl diphosphate synthase; and SPPS, solanesyl diphosphate synthase.

A ML evolutionary tree was built to determine the phylogenetic relationships between the *C. camphora TIDS* family and those found in the other plants (Figure 2). None of the genes apart from those in *S. lycopersicum* and *A. thaliana* has yet been functionally characterized. In order to better distinguish the functional attributes of the *TIDS* genes on each branch, we added several well-characterized TIDS genes when constructing the phylogenetic tree (Supplementary Table S3). The *TIDS* genes in the resulting tree clustered into recently described catalytically distinct subfamilies (Jia and Chen, 2016).

According to enzymatic activity and the relevance of genetic evolution, the subfamily encoding TIDS-e proteins was subdivided into two clades, which contained genes encoding GPPS small subunits I and II. Among them, only one single GPPS small subunit-encoding *C. camphora* gene, *CcTIDS10*, was placed (within the small subunit I clade), indicating that the genome of *C. camphora* only contained GPPS small subunit genes from this class. *CcTIDS6* and *CcTIDS7* were clustered closely with the genes encoding the TIDS-b subfamily of proteins, indicating that these two genes may function as SPPS enzymes.

*CcTIDS1*, *CcTIDS2*, and *CcTIDS8* were found to belong to the TIDS-d subfamily, and therefore, may encode GGPPS, GFPPS, or PPPS enzymes. *CcTIDS3* and *CcTIDS9* were clustered with genes encoding TIDS-a proteins, indicating that they may encode FPPS enzymes. Although, the TIDS-c subfamily is known to contain mostly PPPS (Jia and Chen, 2016), genes encoding functionally characterized GPPS enzymes were included in this clade according to our analysis. Two *CcTIDSs*, *CcTIDS4*, and *CcTTPS5*, were clustered within this subfamily and were more closely related with the known GPPS-encoding genes, indicating that they may possess GPPS activity as well (Figure 2).

### Conserved Motifs, Exon-Intron Structure, and Multiple Gene Alignment Analysis of *CcTIDS* in the Camphor Tree

The evolution of gene families may be accompanied by changes in gene structure, which may provide additional information from which the diversification of gene functions can be inferred (Ye et al., 2017; Cui et al., 2019; Li et al., 2020). For this reason, we further analyzed the organization of the exons and introns of *CcTIDS* genes and compared them with those from *A. thaliana* and *S. lycopersicum*. Our results showed that the *TIDS* genes within the same subgroup mostly exhibited similar arrangements of exons and introns. For instance, *CcTIDS6* and *CcTIDS7*, which were assigned to the TIDS-b-encoding subfamily, possessed six introns each. *CcTIDS1*, *CcTIDS2*, and *CcTIDS8* were all found to contain no introns, indicating that they do not encode GFPPS, which are known to contain two introns, although, they were closely clustered with known GFPPS genes, and belong to the same subfamily (Figure 1). However, it remains unclear whether these genes encode GPPS large subunits or GGPPS proteins.

**Figure 3:**
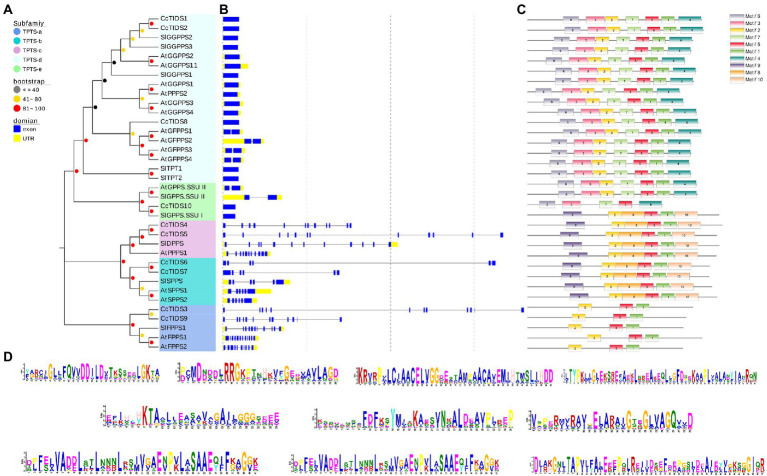
Tran-prenyltransferase protein subfamily analysis based on phylogenetic relationships. **(A)** Intron-exon structures, **(B)** conserved motifs, **(C)** analysis of *C. camphora* (Cc)TIDS, *Arabidopsis thaliana* (At)TIDS, and *Solanum lycopersicum* (Sl)TIDS. **(D)** Legend depicting the protein sequence of the corresponding motif. GPPS, geranyl diphosphate synthase; FPPS, farnesyl diphosphate synthase; GGPPS, geranylgeranyl diphosphate synthase; LSU, large subunit; SSUI/II, small subunit I/II; GFPPS, geranylfarnesyl diphosphate synthase; PPPS, polyprenyl diphosphate synthase; and SPPS, solanesyl diphosphate synthase.

Our analysis on conserved TIDS motifs showed that TIDS belonging to the same subfamily exhibited a similar motif composition and arrangement (Figures 3A–D). Catalytically important conserved motifs were recognized in every identified TIDS sequence after multiple sequence alignment of CcTIDS, *A. thaliana* TIDS (AtTIDS), and *S. lycopersicum* TIDS (SlTIDS). Two common aspartate-rich motifs, the first and second aspartate-rich motif (FARM and SARM, respectively), are known binding sites for DMAPP (Su et al., 2019), and these were present in sequences of all the TIDS sequences except for those of CcTIDS10, AtGPPS small subunit II, and SlGPPS small subunit I.

In addition, two conserved cysteine-rich (CxxxC) motifs were found in CcTIDS10, AtGPPS small subunit II, and SlGPPS small subunit I, and one each in CcTIDS1, CcTIDS2, and CcTIDS8. These CxxxC motifs play important roles in the interaction between the two subunits of the plant heterodimer GPPS. Our data suggest that CcTIDS10 is most likely a GPPS small subunit that forms a heterodimer with CcTIDS1, CcTIDS2, or CcTPS8 as a GPPS large subunit (Supplementary Figure S1).

### Cis-Element Analysis of the TIDS Genes in *C. camphora*

We analyzed cis-elements in the promoter of the *CcTIDS* genes to better understand their potential regulation and function. In total, 343 cis-acting elements were identified, and they were grouped into three categories, which were responsible for plant growth and development, phytohormone responsiveness, and stress responsiveness, according to previous research (Abdullah et al., 2018). Of these, nine belong to the plant growth and development category, of which AAGAA motifs (involved in the endosperm) and AS-1 elements (involved in shoot expression) accounted for the highest proportion (19.05%).

**Figure 4:**
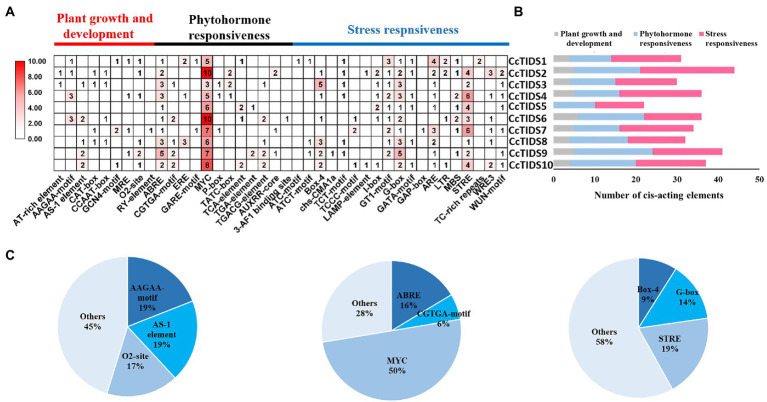
**(A)** The number of cis-acting elements in each *C. camphora (Cc)TIDS* gene, indicated by the intensity of the red color and numbers in the grid. **(B)** Histograms indicating the number of cis-acting elements in each *CcTIDS* gene with functions in plant growth and development, phytohormone responsiveness, or stress responsiveness. **(C)** Pie charts showing the ratio of different cis-acting elements in each structural category.

The greatest proportion of cis-acting regulatory elements related to phytohormone response were myelocytomatosis (MYC) elements that are associated with methyl jasmonate (MeJA), accounting for 50%. All *CcTIDS* genes contained at least five MYC cis-acting elements, suggesting that *CcTIDS* gene expression might be moderated by MeJA. Nearly half of all the cis-acting elements were associated with stress responsiveness (167/343), of which the most common three cis-acting elements were the STRE motif related to stress (19%), G-box (14%), and box 4 (9%), which are associated with responsiveness to light (Figure 4). These results suggest that *CcTIDS* gene expression may be induced or suppressed by MeJA, and these genes may play roles in plant responses to a variety of abiotic stressors.

### Expression Patterns of *CcTIDS* Genes in Different *C. camphora* Tissues

Our sequence alignment, evolution, and gene structure analyses all indicated that the *CcTIDS6* and *CcTIDS7* genes most likely encode SPPS enzymes. Hence, only the eight putative SC-TIDS-encoding genes were included in further analyses of expression patterns and subcellular localization, and an enzymatic assay.

**Figure 5:**
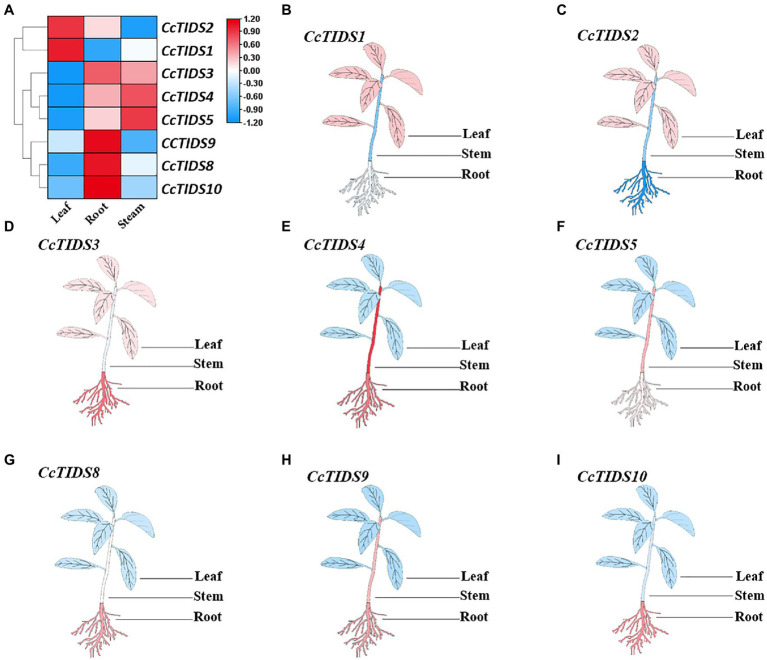
Differential expression of *C. camphora (Cc)TIDS* genes in different tissues by transcriptome and quantitative real-time PCR (qRT-PCR). **(A)** FPKM value of the RNA sequencing data for each gene in leaf, root, and stem tissues. **(B–I)** Relative expression of each gene in the different plant tissues as calculated by qRT-PCR, with red and blue representing high and low expression levels, respectively.

To identify potential roles for *CcTIDS* genes in different tissues of *C. camphora*, we established a transcriptome database based on RNA sequencing of the leaves, roots, and stems (Accession number: PRJNA747104). qRT-PCR was used to verify these transcriptome data. We found that the expression profiles of *CcTIDS* genes were tissue-specific (Figure 5). For example, *CcTIDS1* and *CcTIDS2* were upregulated in the leaf tissue compared with the root and stem, whereas the expression level of *CcTIDS3*, *9*, *8*, and *10* were upregulated in the root compared with the other tissues. This may indicate that there are functional differences in *CcTIDS* according to the different *C. camphora* organs from which the tissue was derived.

### Subcellular Localization of Putative SC-TIDS Proteins

**Figure 6:**
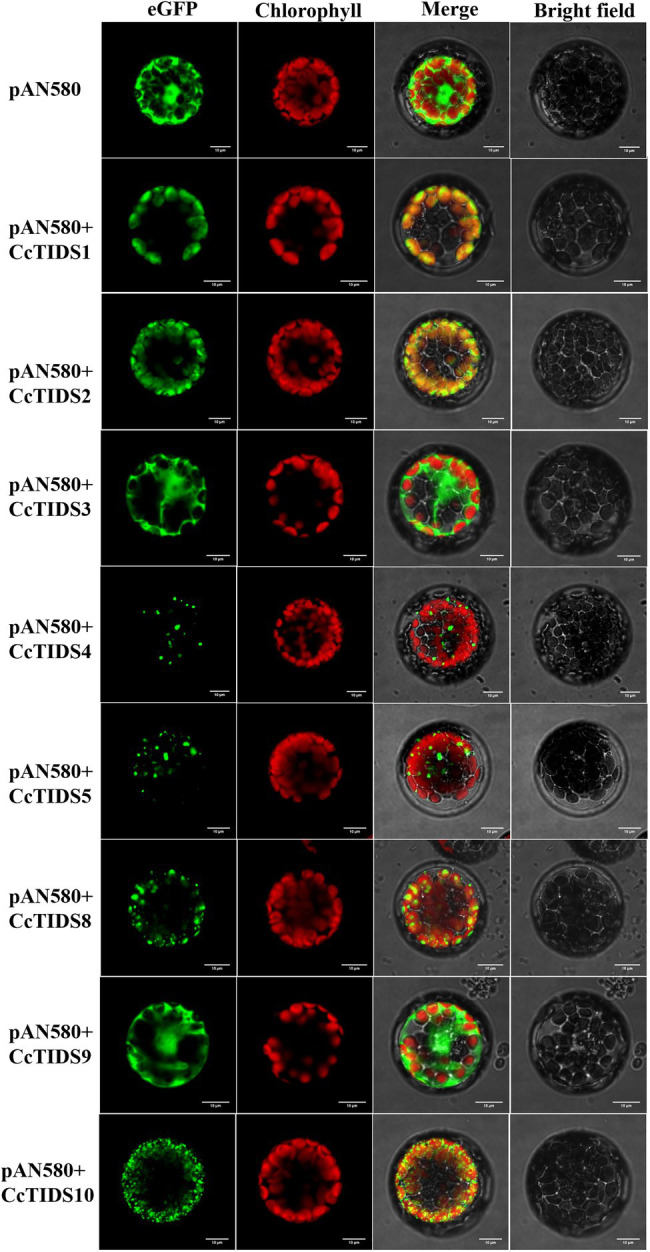
Subcellular localization of eight putative *C. camphora* trans-prenyltransferase (CcTIDS) enzymes in *A. thaliana* protoplasts. eGFP, green fluorescent protein fluorescence image; chlorophyll, chlorophyll autofluorescence image; bright-field, transmission image; merged, all channels (eGFP, chlorophyll, and bright-field) combined.

Subcellular localization predictions using four different online software programs revealed that CcTIDS1, 2, 8, and 10 are likely to be located in plastids. However, the results for CcTIDS3, 4, 5, and 9 were inconsistent (Table 1). In order to obtain exact subcellular location information for each candidate CcTIDS, we analyzed expression patterns using fluorescence microscopy (Figure 6). Consistent with all prediction programs, the GFP signals for CcTIDS1, 2, 8, and 10 were localized mainly within chloroplasts. Fluorescently tagged CcTIDS3 and CcTIDS9 proteins were both localized in the cytoplasm, while fluorescent signals for CcTIDS4 and CcTIDS5 recombinant proteins indicated that they may be located within mitochondria.

### Heterologous Expression and *in vitro* Functional Characterization of the SC-TIDS Proteins

*In vitro* enzymatic assays were conducted using recombinant proteins extracted and purified from *E. coli* expression strains. The large subunit of CrGPPS was found to catalyze IPP and DMAPP to form geranyl diphosphate (GPP) and geranylgeranyl diphosphate (GGPP), whereas the small subunit of CrGPPS was found to be inactive alone. However, when both subunits were co-incubated, they catalyzed the formation of GPP (Supplementary Figure S2). This is accordance with the results reported in previous research, indicating that the method is valid for the verification of SC-TIDS *in vitro* enzymatic activity (Rai et al., 2013).

To characterize the two putative CcGPPS proteins (CcTIDS4 and 5), full-length and truncated versions of CcGPPSs were both analyzed. However, full-length recombinant CcGPPS protein was found to be completely insoluble. Therefore, soluble protein that was purified from the truncated version of each CcGPPS was used in the *in vitro* enzymatic activity assays. Unexpectedly, no catalytic products were detected for the truncated versions of both CcTIDS4 and CcTIDS5 under the conditions used here (Table 2).

**Table 2:** Functionally characterized enzymes in this study.

Genes	Full-length ORF/bp	Variants studied	Accepted substrates	Products	Rename
CcTIDS1	1,149	Ala62-end	DMAPP+IPP	GGPP	CcGGPPS1
CcTIDS2	1,161	Asp65-end	DMAPP+IPP	GGPP	CcGGPPS2
CcTIDS3	1,050	Full length	DMAPP+IPP	FPP	CcFPPS1
			GPP+IPP	FPP	
CcTIDS4	1,263	Full length	No activity	-	-
	1,263	M101-end	No activity	-	
CcTIDS5	1,284	Full length	No activity	-	-
	1,284	M108-end	No activity	-	
CcTIDS8	1,149	Lys56-end	No activity	-	CcGPPS.LSU
CcTIDS9	1,092	Full length	DMAPP+IPP	FPP	CcFPPS2
			GPP+IPP	FPP	
CcTIDS10	894	Asn65-end	No activity	-	CcGPPS.SSU
CcTIDS8&CcTIDS10	1,149&894	Lys56-end&Asn65-end	DMAPP+IPP	GPP	

**Figure 7:**
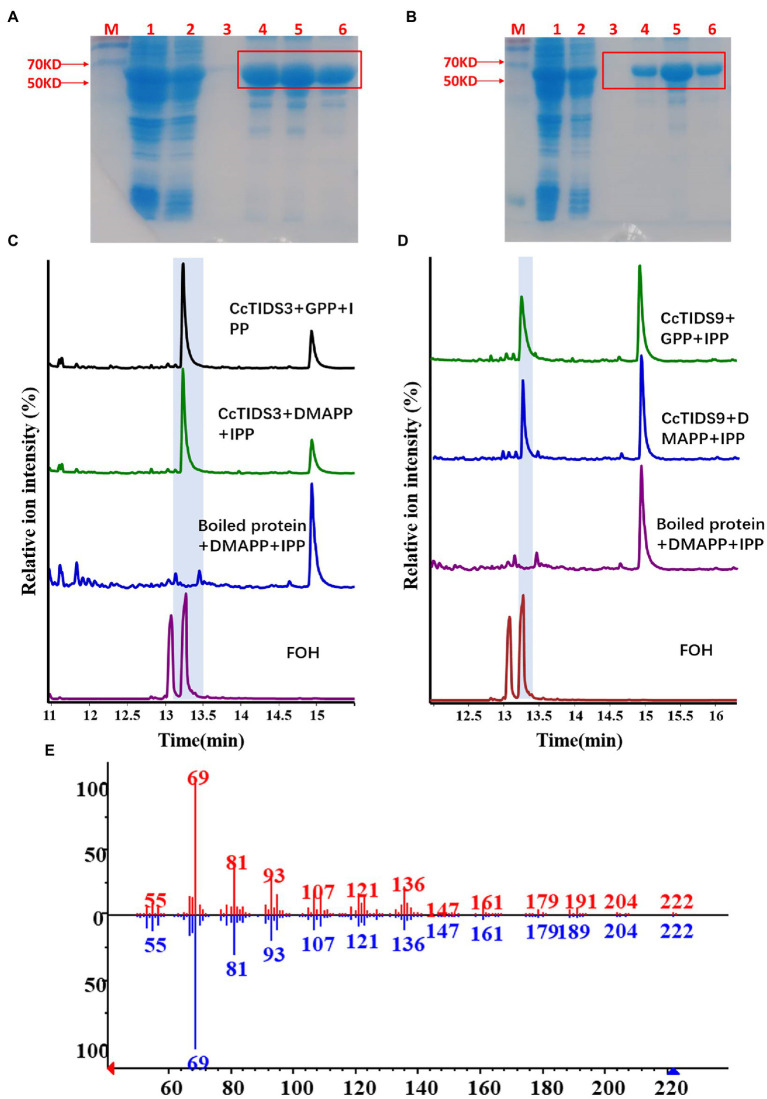
Gas chromatography–mass spectrometry (GC–MS) profile showing *in vitro* reaction products of *C. camphora* trans-prenyltransferases CcTIDS3 and CcTIDS9, where different prenyldiphosphates were used as substrates. Expression and purification of **(A)** CcTIDS3 and **(B)** CcTIDS9 recombinant protein from *Escherichia coli* Rosetta (DE3) harboring pET32a(+)–CcTIDS3/CcTIDS9, where lane 1 shows the total protein after induction, 2 shows the soluble protein, and 3–6 shows the purified CcTIDS3 or CcTIDS9 recombinant protein from the first to the fourth collected tube. The GC–MS chromatogram of the reaction products generated by **(C)** CcTIDS3 and **(D)** CcTIDS9 and the acid hydrolysis products of farnesol (FOH) standards. **(E)** Mass spectra of farnesol standard (shown in red) and products in the NIST14/Wiley275 library (blue). GPP, geranyl diphosphate; DMAPP, dimethylallyl diphosphate; and IPP, isopentenyl diphosphate.

Two full-length putative CcFPPS proteins (CcTIDS 3 and 9) lacking a transit peptide were generated. As expected, both recombinant proteins exhibited FPPS activity, producing FPP as the unique product, indicating that these two TIDS proteins are FPP synthases. Therefore, we reclassified these two TIDS proteins as CcFPPS1 and CcFPPS2 (Figure 7).

**Figure 8:**
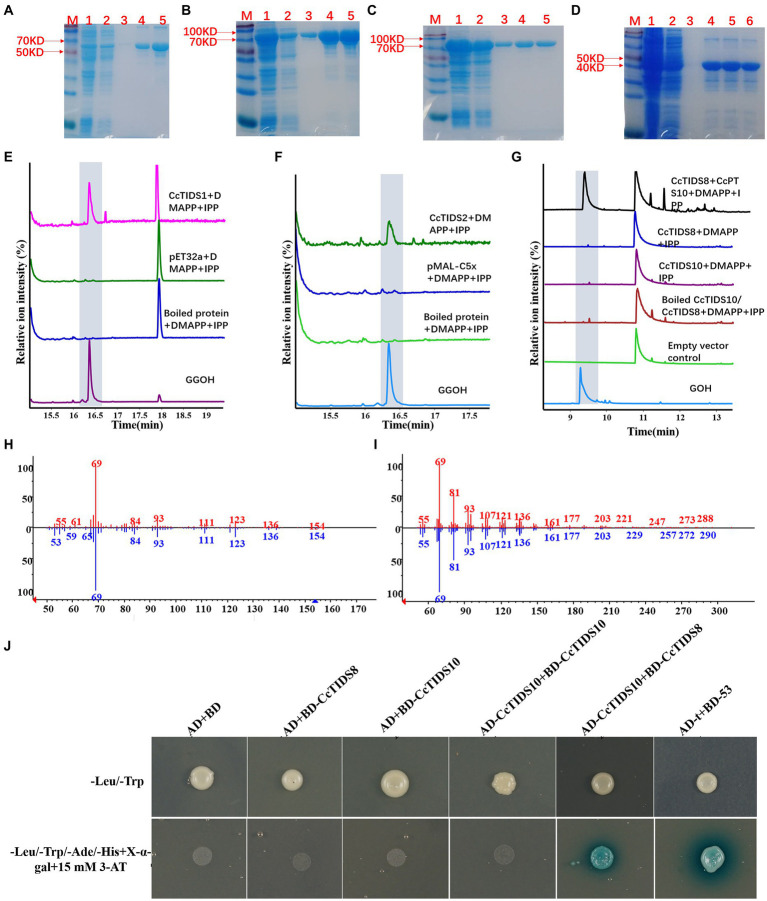
Gas chromatography–mass spectrometry profile showing *in vitro* reaction products of *C. camphora* trans-prenyltransferases CcTIDS1, CcTIDS2, CcTIDS8, and CcTIDS9, where different prenyldiphosphates were used as substrates. Expression and purification of **(A)** CcTIDS1, **(B)** CcTIDS2, **(C)** CcTIDS8, and **(D)** CcTIDS10 recombinant protein from *E. coli* Rosetta (DE3) harboring pET32a(+)–CcTIDS1/CcTIDS10 or pMAL-C5x–CcTIDS2/CcTIDS8, where lane 1 shows the total protein after induction, 2 shows the soluble protein, 3–6 show the purified CcTIDS1 or CcTIDS2, CcTIDS8, or CcTIDS10 recombinant protein from the first to the fourth collected tube. The GC–MS chromatogram of the reaction products generated by **(E)** CcTIDS8 and CcTIDS10, **(F)** CcTIDS1, and **(G)** CcPTS2 and the acid hydrolysis products of geraniol (GOH) and gerylgeraniol (GGOH) standards. **(H)** Mass spectra of GOH standard (red) and products in the NIST14/Wiley275 library (blue). **(I)** Mass spectra of the GGOH standard (red) and products in the NIST14/Wiley275 library (blue). **(J)** Confirmation of the interaction between CcTIDS8 and CcTIDS10 using a yeast two-hybrid system, where blue color indicates an interaction. Yeast cells harboring both constructs were spotted on synthetic dropout (SD) medium lacking Ade, His, Leu, and Trp (SD/−4) to test for protein interactions. AD: pGADT7; BD: pGBDT7; AD-T: pGADT7::T; BD-53: pGBKT7-53. Cells co-transformed with pGADT7::T and pGBKT7-53 were included as a positive control, and pGADT7 and pGBDT7 as a vector control.

It was predicted that three putative CcGGPPS proteins (CcTIDS1, 2, and 8) and one putative CcGPPS small subunit (CcTIDS10) contained a transit peptide. Therefore, truncated versions of these four proteins were fused to His or MBP tags, resulting in the formation of soluble protein. The activity of these four soluble proteins was detected in all substrate combinations. As a result, CcTIDS1 and 2 emitted a prominent chromatographic signal corresponding to geranylgeraniol (C20), demonstrating that CcTIDS1 and 2 function individually as *bona fide* GGPPS enzymes. Hence, these two proteins were renamed CcGGPPS1 and CcGGPPS2 (Figures 8A–F). In contrast, CcTIDS8 and 10 did not exhibit any chromatographic peak signal for farnesol (C15), geraniol (C10), or geranylgeraniol (C20), suggesting that these proteins are inactive alone (Figure 8G).

### CcTIDS8 Interacts With CcTIDS10 to Generate GPP

Because CcTIDS10 was identified as the only CcGPPS small subunit, we determined whether this protein was able to interact with CcGGPPS1/2 or CcTIDS8 to form active heterodimers. Intriguingly, when CcTIDS10 was co-incubated with CcTIDS8, but not CcGGPPS1 or 2, GPP was detected as a sole product (Figures 8G-I).

A yeast two-hybrid system (Y2H) was used to confirm the interaction of CcTIDS10 with CcTIDS8. As expected, CcTIDS10 was able to interact with CcTIDS8 and itself (Figure 8J). These results, along with the co-localization of CcTIDS10 and CcTIDS8 in the plastid, confirmed that the inactive CcTIDS10 and CcTIDS8 interact to form heteromeric functional GPPS, generating GPP as the sole product. Thus, CcTIDS10 was designated as CcGPPS.small subunit (SSU), whereas CcTIDS8 was renamed as CcGPPS.large subunit (LSU).

## Discussion

In this study, we reported the characterization of the *C. camphora* TIDS gene family for the first time. Of the 10 identified *TIDS* genes, three (*CcGGPPS1*, *CcGGPPS2*, and *CcGPPS.LSU*) were clustered within the subfamily encoding TIDS-d enzymes, while one single gene, *CcTIDS10*, was closely related to the subfamily encoding TIDS-e enzymes. Apart from those in gymnosperms, TIDS proteins within the TIDS-d and TIDS-e subfamily have been classified as GGPPS paralogs in previous studies. As a result, many GGPPS homologs were predicted from the plant genomes (Coman et al., 2014).

The expansion of the GGPPS family in *A. thaliana* occurred at distinct evolutionary time points through different duplication mechanisms, including whole gene, tandem, and segmental genome duplications (Coman et al., 2014). The evolution of this *A. thaliana* GGPPS family likely involved neofunctionalization (with the duplicated gene developing a function that was not present in the ancestral gene), subfunctionalization (in which the duplicated and ancestral genes retain different parts of the original function of the ancestral gene), and pseudogenization (loss of function; Coman et al., 2014). Based on our fragment duplication and functional differentiation results for *CcGGPPS1*, *CcGGPPS2*, and *CcGPPS.LSU*, and the phylogenetic tree we constructed, these three genes may have been derived from the same ancestor, having undergone neo- or subfunctionalization.

Thus far, homodimeric GPPS has been functionally characterized in only a few plant species (Burke et al., 2004; Hsiao et al., 2008; Schmidt and Gershenzon, 2008; Gutensohn et al., 2013; Rai et al., 2013; Adal and Mahmoud, 2020). We found that the *C. camphora* genome contained two putative homodimeric *GPPS* genes (*CcTIDS4* and *CcTIDS5*). Our evolutionary analysis suggested that these two genes were related to those encoding the functionally characterized homodimeric GPPS from *A. thaliana*, and subcellular localization experiments indicated that the two proteins encoded by these genes might localize to the mitochondria, consistent with earlier reports on *C. roseus* (Rai et al., 2013). However, there was no observable GPPS activity associated with the proteins *in vitro*.

Instead, CcGPPS.SSU (CcTIDS10) and CcGPPS.LSU (CcTIDS8), both identified in our study, were found to interact with one another to form a heterodimeric CcGPPS that produced GPP from IPP and DMAPP substrates *in vitro*. These proteins were found to be inactive alone. This is consistent with previous reports in both *Mentha*×*piperita* subunits (Burke et al., 2004), both *Lavendula*×*intermedia* subunits (Adal and Mahmoud, 2020), and *A. thaliana* GPPS.SSU2 (Wang and Dixon, 2009).

CcGPPS.SSU and CcGPPS.LSU were primarily expressed in roots compared with stems and leaves. Furthermore, both CcGPPS.SSU and CcGPPS.LSU were found to reside in plastids, which are the known site of monoterpene biosynthesis (Magnard et al., 2015; Yin et al., 2017). These results suggest that heteromeric CcGPPS likely participates in the biosynthesis of GPP in *C. camphora*.

Interestingly, all *CcTIDS* genes had orthologs of greater than 90% identity with the *C. micranthum* genome, with the sole exception of *CcGPPS.SSU* (Supplementary Figure S3). *Cinnamomum micranthum*, known as the stout camphor tree, is the sister species of *C. camphora*. The genome of *C. micranthum* with a contig N_50_ of 0.9Mb has been published. However, contig N_50_ of the genome of *C. camphora* is 23.89Mb (data not shown), indicating that the assembly quality of the *C. camphora* genome is significantly better than that of the *C. micranthum* genome. This lower quality may explain why the GPPS.SSU gene is missing from the *C. micranthum* genome.

In addition, although, CcTIDS4 and CcTIDS5 did not possess GPPS activity in our study, they may perform different functions. For example, At2g34630 from *A. thaliana* was initially classified as a homodimeric GPPS (Bouvier et al., 2000), but further study indicated that *At2g34630* silencing did not affect the production of monoterpenes, and At2g34630 was subsequently identified as a PPPS (Hsieh et al., 2011). Therefore, the functions of CcTIDS4 and 5 should be investigated in future studies.

**Figure 9:**
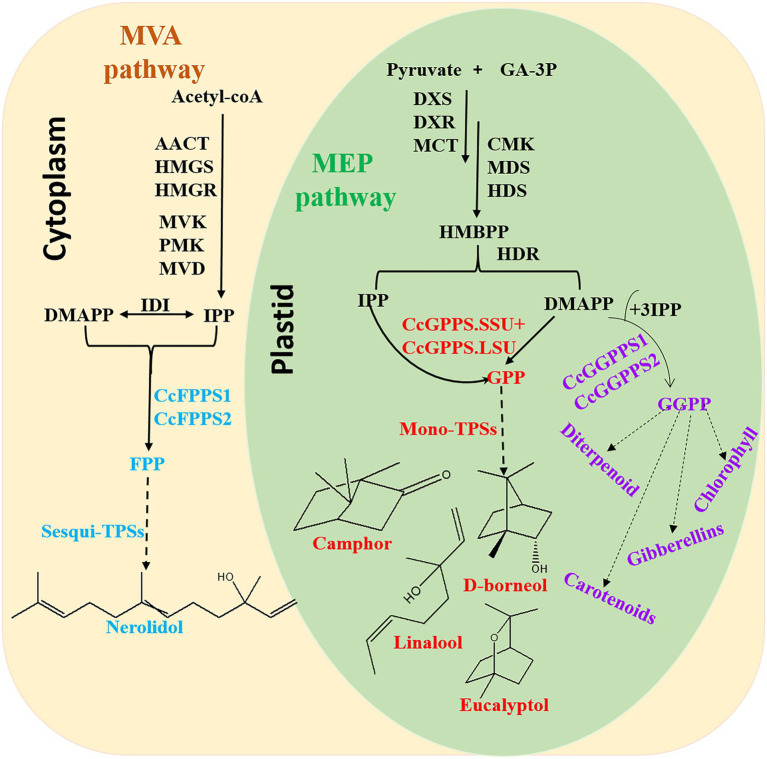
Overview of the terpenoid biosynthesis pathway in *C. camphora*. AACT, acetoacetyl-coenzyme A thiolase; HMGS, 3-hydroxy-3-methylglutaryl coenzyme A synthase; HMGR, 3-hydroxy-3-methylglutaryl coenzyme A reductase; MVK, mevalonate kinase; PMK, 5-phospho mevalonate kinase; MVD, mevalonate diphosphate decarboxylase; IDI, isopentenyl diphosphate isomerase; FPPS, farnesyl diphosphate synthase; DXS,1-deoxy-D-xylulose-5-phosphate synthase; DXR,1-deoxy-D-xylulose-5-phosphate reductoisomerase; MCT, 2-C-methyl-D-erythritol-4-(cytidyl-5-diphosphate) transferase; CMK, 4-(cytidine50-diphospho)-2-C-methyl-D-erythritol kinase; MDS, 2-C-methyl-D-erythritol 2,4-cyclodiphosphate synthase; HDS,1-hydroxy-2-methyl-2-(E)-butenyl-4-diphosphate synthase; and HDR, 1-hydroxy-2-methyl-2-(E)-butenyl-4-diphosphate reductase.

In conclusion, this is the first comprehensive and systematic genome-wide analysis of TIDS gene families in *C. camphora*. In total, 10 TIDS genes in the borneol chemotype of *C. camphora* were identified. These genes likely expanded through segmental duplication events, and expression was found to respond to multiple abiotic stressors *via* cis-acting elements in the promoter region. Eight putative SC-TIDS were identified, six of which were catalytically active, including a heteromeric GPPS (composed of CcGPPS.SSU and CcGPPS.LSU), two CcFPPS (CcTIDS3 and 9), and two CcGGPPS (CcTIDS1 and 2), which catalyzed the biosynthesis of GPP, FPP, and GGPP, respectively (Figure 9).

Finally, *CcTIDS3*, *8*, *9*, and *10* were more active in roots compared with stems and leaves, which were confirmed by both transcriptome analysis and qRT-PCR experiments. These novel insights provide the basis for further investigation of the TIDS family in *C. camphora*, and a good foundation from which to develop a metabolic engineering approach to increase production of pharmacologically valuable essential oils from the camphor tree.

## Data Availability Statement

The original contributions presented in the study are included in the article/Supplementary Material, further inquiries can be directed to the corresponding authors.

## Author Contributions

ZY conceived, designed, and performed the experiments, analyzed the data, prepared the figures and tables, and authored and reviewed drafts of the paper. CX and LL conceived, designed, and performed the experiments, analyzed the data, and authored and reviewed drafts of the paper. SL, YH, and WA conceived, designed, and performed the experiments, and authored and reviewed drafts of the paper. SH and XZ conceived and designed the experiments, analyzed the data, prepared the figures and tables, and authored and reviewed drafts of the paper. All authors contributed to the article and approved the submitted version.

## Funding

This research was supported by the National Natural Science Foundation of China (grant number: 81903741).

## Conflict of Interest

The authors declare that the research was conducted in the absence of any commercial or financial relationships that could be construed as a potential conflict of interest.

## Publisher’s Note

All claims expressed in this article are solely those of the authors and do not necessarily represent those of their affiliated organizations, or those of the publisher, the editors and the reviewers. Any product that may be evaluated in this article, or claim that may be made by its manufacturer, is not guaranteed or endorsed by the publisher.

## Footnotes

^1^ https://www.arabidopsis.org/index.jsp

^2^ https://solgenomics.net/organism/Solanum_lycopersicum/genome/

^3^ https://blast.ncbi.nlm.nih.gov/Blast.cgi

^4^ https://web.expasy.org/protparam

^5^ https://meme-suite.org/meme/tools/meme

^6^ http://www.cbs.dtu.dk/services/ChloroP/

^7^ http://www.cbs.dtu.dk/services/TargetP/

^8^ https://wolfpsort.hgc.jp/

^9^ http://www.csbio.sjtu.edu.cn/bioinf/plant-multi/
