# Supplementary material for: Genome-Wide Identification and Functional Characterization of the Trans-Isopentenyl Diphosphate Synthases Gene Family in Cinnamomum camphora
Source: Front Plant Sci. 2021 Sep 13;12:708697. doi: 10.3389/fpls.2021.708697 (PMC8475955; doi:10.3389/fpls.2021.708697)
Supplement: Supplementary file 1 [file Data_Sheet_1.docx]

**Table S1** Numbers of TIDS subfamilies in the 10 genomes major seed plant lineages

| Species | TIDSa | TIDSb | TIDSc | TIDSd | TIDSe | Sum |
| --- | --- | --- | --- | --- | --- | --- |
| *Arabidopsis thaliana* | 2 | 2 | 1 | 10 | 1 | 16 |
| *Populus trichocarpa* | 1 | 4 | 2 | 5 | 2 | 14 |
| *Solanum lycopersicum* | 1 | 1 | 1 | 5 | 2 | 10 |
| *Cinnamomum micranthum* | 2 | 2 | 2 | 3 | 0 | 10 |
| *Cinnamomum camphora* | 2 | 2 | 2 | 4 | 1 | 10 |
| *Oryza sativa* | 5 | 2 | 2 | 2 | 1 | 12 |
| *Zea mays* | 3 | 2 | 2 | 3 | 2 | 12 |
| *Picea abies* | 0 | 0 | 0 | 7 | 0 | 7 |
| *Amborella trichopoda* | 1 | 1 | 1 | 2 | 1 | 6 |
| *Selaginella moellendorffii* | 2 | 1 | 1 | 1 | 1 | 6 |
| *Physcomitrella patens* | 1 | 2 | 2 | 2 | 0 | 7 |

**Table S2** TIDS identified in the 10 genomes major seed plant lineages

| *Species* | Gene ID | Subfamily |
| --- | --- | --- |
| *Cinnamomum camphora* | evm.model.LG01.0506 | TIDS-d |
|  | evm.model.LG01.2465 | TIDS-d |
|  | evm.model.LG04.0985 | TIDS-a |
|  | evm.model.LG04.2634 | TIDS-c |
|  | evm.model.LG05.1976 | TIDS-c |
|  | evm.model.LG06.0936 | TIDS-b |
|  | evm.model.LG08.1082 | TIDS-b |
|  | evm.model.LG11.0380 | TIDS-d |
|  | evm.model.LG11.1115 | TIDS-a |
|  | evm.model.LG11.1288 | TIDS-e |
| *Cinnamomum micranthum* | CKAN_00054800 | TIDS-d |
|  | CKAN_00258700 | TIDS-d |
|  | CKAN_01052000 | TIDS-a |
|  | CKAN_01215200 | TIDS-c |
|  | CKAN_01230400 | TIDS-c |
|  | CKAN_01471200 | TIDS-b |
|  | CKAN_01525000 | TIDS-b |
|  | CKAN_01813100 | TIDS-d |
|  | CKAN_02433600 | TIDS-a |
|  | CKAN_02504500 | TIDS-d |
|  | AT4G38460.1 | TIDS-e |
|  | AT5G47770.1 | TIDS-a |
|  | AT4G17190.1 | TIDS-a |
|  | AT1G49530.1 | TIDS-d |
|  | AT2G18620.1 | TIDS-d |
|  | AT2G18640.1 | TIDS-d |
|  | AT2G23800.1 | TIDS-d |
|  | AT4G36810.1 | TIDS-d |
|  | AT3G14530.1 | TIDS-d |
|  | AT3G14550.1 | TIDS-d |
|  | AT3G29430.1 | TIDS-d |
|  | AT3G32040.1 | TIDS-d |
|  | AT2G34630.2 | TIDS-c |
|  | AT3G20160.1 | TIDS-d |
|  | AT1G78510.1 | TIDS-c |
|  | AT1G17050.1 | TIDS-c |
| *Solanum lycopersicum* | Solyc12g015860 | TIDS-a |
|  | Solyc11g011240 | TIDS-d |
|  | Solyc04g079960 | TIDS-d |
|  | Solyc07g061990 | TIDS-b |
|  | Solyc08g023470 | TIDS-c |
|  | Solyc02g085700 | TIDS-d |
|  | Solyc02g085710 | TIDS-d |
|  | Solyc02g085720 | TIDS-d |
|  | Solyc07g064660 | TIDS-e |
|  | Solyc09g008920 | TIDS-e |
| *Populus trichocarpa* | Potri.006G003400.1 | TIDS-a |
|  | Potri.007G031100.1 | TIDS-d |
|  | Potri.005G127100.1 | TIDS-d |
|  | Potri.015G043400.1 | TIDS-e |
|  | Potri.009G139600.1 | TIDS-e |
|  | Potri.017G124700.1 | TIDS-d |
|  | Potri.017G124600.1 | TIDS-d |
|  | Potri.004G090600.1 | TIDS-d |
|  | Potri.010G138800.1 | TIDS-c |
|  | Potri.006G135300.1 | TIDS-c |
|  | Potri.001G380500.1 | TIDS-b |
|  | Potri.011G101200.1 | TIDS-b |
|  | Potri.011G101400.1 | TIDS-b |
|  | Potri.011G099500.1 | TIDS-b |
| *Oryza sativa* | Os01g14630 | TIDS-d |
|  | Os01g50050 | TIDS-a |
|  | Os01g50760 | TIDS-a |
|  | Os02g44780 | TIDS-e |
|  | Os04g56210 | TIDS-a |
|  | Os04g56230 | TIDS-a |
|  | Os05g46580 | TIDS-a |
|  | Os05g50550 | TIDS-b |
|  | Os06g46450 | TIDS-c |
|  | Os07g39270 | TIDS-d |
|  | Os08g09370 | TIDS-c |
|  | Os12g17320 | TIDS-b |
| *Zea mays* | Zm00001d021929_P001 | TIDS-d |
|  | Zm00001d006678_P001 | TIDS-d |
|  | Zm00001d027694_P001 | TIDS-b |
|  | Zm00001d039142_P001 | TIDS-b |
|  | Zm00001d043727_P001 | TIDS-a |
|  | Zm00001d011673_P001 | TIDS-a |
|  | Zm00001d008370_P001 | TIDS-a |
|  | Zm00001d009431_P001 | TIDS-d |
|  | Zm00001d051416_P001 | TIDS-e |
|  | Zm00001d014367_P001 | TIDS-c |
|  | Zm00001d017540_P001 | TIDS-e |
|  | Zm00001d014369_P002 | TIDS-c |
| *Selaginella moellendorffii* | 177791\|PACid:15406521 | TIDS-b |
|  | 133501\|PACid:15408951 | TIDS-e |
|  | 127038\|PACid:15409712 | TIDS-a |
|  | 173042\|PACid:15411775 | TIDS-a |
|  | 231193\|PACid:15414701 | TIDS-d |
|  | 415177\|PACid:15418076 | TIDS-c |
| *Physcomitrella patens* | Pp3c24_18700V3.1.p | TIDS-a |
|  | Pp3c21_14800V3.1.p | TIDS-d |
|  | Pp3c3_31610V3.1.p | TIDS-c |
|  | Pp3c27_1880V3.1.p | TIDS-b |
|  | Pp3c8_7380V3.1.p | TIDS-c |
|  | Pp3c18_14700V3.1.p | TIDS-d |
|  | Pp3c16_16550V3.1.p | TIDS-b |
| *Picea abies* | MA_1011718g0010 | TIDS-d |
|  | MA_10429756g0010 | TIDS-d |
|  | MA_138828g0010 | TIDS-d |
|  | MA_194998g0010 | TIDS-d |
|  | MA_543467g0010 | TIDS-d |
|  | MA_888237g0010 | TIDS-d |
|  | MA_895626g0010 | TIDS-d |
| *Amborella trichopoda* | evm_27.model.AmTr_v1.0_scaffold00061.202 | TIDS-a |
|  | evm_27.model.AmTr_v1.0_scaffold00086.58 | TIDS-b |
|  | evm_27.model.AmTr_v1.0_scaffold00092.58 | TIDS-c |
|  | evm_27.model.AmTr_v1.0_scaffold00566.1 | TIDS-d |
|  | evm_27.model.AmTr_v1.0_scaffold00059.110 | TIDS-d |
|  | evm_27.model.AmTr_v1.0_scaffold00001.167 | TIDS-e |

**Table S3** Functional characterized TIDS which were used for constructing the evolutionary tree

| Gege | Accession number | Species |
| --- | --- | --- |
| AgGPPS2 | AAN01134.1 | *Abies grandis* |
| AgGPPS1 | AAN01133.1 | *Abies grandis* |
| AgGPPS3 | AAN01135.1 | *Abies grandis* |
| CaGPPS | ATZ76916.1 | *Camptotheca acuminata* |
| EpFPPS | ACN63187.1 | *Euphorbia pekinensis* |
| CrFPPS | ADO95193.1 | *Catharanthus roseus* |
| LiFPPS | QHN60322.1 | *Lavandula x intermedia* |
| GbFPPS | AAR27053.1 | *Ginkgo biloba* |
| LiGPPSGPPS.LSU | QHN60320.1 | *Lavandula x intermedia* |
| LiGPPS.SSUII | QHN60319.1 | *Lavandula x intermedia* |
| LiGPPS.SSU | AGH33891.1 | *Lavandula x intermedia* |
| QrGPPS | CAC20852.1 | *Quercus robur* |
| CsGPPS | CAC16851.1 | *Citrus sinensis* |
| CrGPPS | ACC77966.1 | *Catharanthus roseus* |
| CrLSU | AEI53622.1 | *Catharanthus roseus* |
| TwGGPPS1 | AKQ99275.1 | *Tripterygium wilfordii* |
| TwGGPPS7 | QFU96146.1 | *Tripterygium wilfordii* |
| TwGGPPS8 | QFU96147.1 | *Tripterygium wilfordii* |
| HlLSU | ACQ90682.1 | *Humulus lupulus* |
| MpLSU | ABW86879.1 | *Mentha x piperita* |
| AmLSU | AAS82860.1 | *Antirrhinum majus* |
| HlSSU | ACQ90681.1 | *Humulus lupulus* |
| MpSSU | ABW86880.1 | *Mentha x piperita* |
| AmSSU | AAS82859.1 | *Antirrhinum majus* |
| CrGPPS.SSU | JX417184 | *Catharanthus roseus* |
| TwSSUI | AKU77012.1 | *Tripterygium wilfordii* |
| TwSSUII | AKU77009.1 | *Tripterygium wilfordii* |

**Table S4**  Primers used in this article

| 引物名称 | 引物序列（5^/^-3^/^） |
| --- | --- |
| CcTIDS1F | ATGGCTGCCATCCTAAACCATT |
| CcTIDS1R | CTACTGTGCACAGCGAGTGGC |
| CcTIDS2F | ATGCTCCAAAAGGCGAATTCAG |
| CcTIDS2R | TCAACGACAAAAAGTAAATGAAGTG |
| CcTIDS3F | ATGGCTGCGGCTTCAAATG |
| CcTIDS3R | CTACTTTTGCCTCTTGTAGATCTTGC |
| CcTIDS4F | ATGCCACTGAGATGGGCAATG |
| CcTIDS4R | TCATTTTGTTCTTGTAATGACTATCTGGG |
| CcTIDS5F | ATGCCTCTCAGATGGGCTCTC |
| CcTIDS5R | TCACTTCGTTCTTGTAATGACTCTG |
| CcTIDS8R | TTAGTTCTGTCTGTAAGCAATGTAATTTGC |
| CcTIDS9F | ATGGCTGCTGCTGCTGCT |
| CcTIDS9R | TTACTTCTGCCTCTTGTAAATCTTGC |
| CcTIDS10F | ATGACGAAATCATTTGCTACTGTTC |
| CcTIDS10R | TTACCTTTGACCTTCCATAAATTGC |
| pET32a-CcTIDS1F | gctgatatcggatccgaattcATGGATGCCCACCTTGCA |
| pET32a-CcTIDS1R | gcaagcttgtcgacggagctcCTAGTTCTGTCTATAAGCAATATAATTCGCTAATGAAATCAG |
| pET32a-CcTIDS2F | gctgatatcggatccgaattcATGGCAACCCACCTTGCAAA |
| pET32a-CcTIDS2R | gcaagcttgtcgacggagctcTTAGTTCTGTCTGTAAGCAATGTAATTTGCCAA |
| pET32a-CcTIDS3F | gctgatatcggatccgaattcATGGCTGCGGCTTCAAATG |
| pET32a-CcTIDS3R | gcaagcttgtcgacggagctcCTACTTTTGCCTCTTGTAGATCTTGC |
| pET32a-CcTIDS4F | gctgatatcggatccgaattcTCCAAAACGAGATTTTACAGAGGA |
| pET32a-CcTIDS4R | gcaagcttgtcgacggagctcTCATTTTGTTCTTGTAATGACTATCTGG |
| pET32a-CcTIDS5F | gctgatatcggatccgaattcGCCTCAAAGGTTTTTGGTGGC |
| pET32a-CcTIDS5R | gcaagcttgtcgacggagctcTCACTTCGTTCTTGTAATGACTCTGTG |
| pET32a-CcTIDS8F | gctgatatcggatccgaattcATGAAAAGGGCACTAAAGACCCC |
| pET32a-CcTIDS8R | gcaagcttgtcgacggagctcCTAGTTGTTTCTGCAAGCAATGAAATCTGT |
| pET32a-CcTIDS9F | gctgatatcggatccgaattcATGGCTGCTGCTGCTGCTGCTG |
| pET32a-CcTIDS9R | gcaagcttgtcgacggagctcTTACTTCTGCCTCTTGTAAATCTTGC |
| pET32a-CcTIDS10F | gctgatatcggatccgaattcATGGCCCGCCTCAAGC |
| pET32a-CcTIDS10R | gcaagcttgtcgacggagctcCTACTGTGCACAGCGAGTGG |
| pMAL-C5X-CcTIDS1F | ccatgggcggccgcgatatcATGGATGCCCACCTTGCA |
| pMAL-C5X-CcTIDS1R | tgcagggaattcggatccCTAGTTCTGTCTATAAGCAATATAATTCGCTAATGAAATCAG |
| pMAL-C5X-CcTIDS8F | atgggcggccgcgatatcATGAAAAGGGCACTAAAGACCCC |
| pMAL-C5X-CcTIDS8R | cagggaattcggatccCTAGTTGTTTCTGCAAGCAATGAAATCTGT |
| pAN580-CcTIDS1F | ttaagtccggagctagctctagaATGGCTTCTTTTGCTTTGATTGCTCA |
| pAN580-CcTIDS1R | tcgcccttgctcaccatggatccGTTCTGTCTATAAGCAATATAATTCGCTAATGAAATCAGAG |
| pAN580-CcTIDS2F | ttaagtccggagctagctctagaATGGCTGCTTTCGCTTTGAT |
| pAN580-CcTIDS2R | tcgcccttgctcaccatggatccGTTCTGTCTGTAAGCAATGTAATTTGCCAA |
| pAN580-CcTIDS3F | ttaagtccggagctagctctagaATGGCTGCGGCTTCAAATG |
| pAN580-CcTIDS3R | tcgcccttgctcaccatggatccCTTTTGCCTCTTGTAGATCTTGCCCA |
| pAN580-CcTIDS4F | ttaagtccggagctagctctagaATGCCACTGAGATGGGCAATG |
| pAN580-CcTIDS4R | tcgcccttgctcaccatggatccTTTTGTTCTTGTAATGACTATCTGGGTAAGATCAACAAG |
| pAN580-CcTIDS5F | ttaagtccggagctagctctagaATGCCTCTCAGATGGGCTCT |
| pAN580-CcTIDS5R | tcgcccttgctcaccatggatccCTTCGTTCTTGTAATGACTCTGTGAGTAAGAT |
| pAN580-CcTIDS8F | ttaagtccggagctagctctagaATGGGTTCTTCAGTGCATCTAGC |
| pAN580-CcTIDS8R | tcgcccttgctcaccatggatccGTTGTTTCTGCAAGCAATGAAATCTGTCA |
| pAN580-CcTIDS9F | ttaagtccggagctagctctagaATGGCTGCTGCTGCTGC |
| pAN580-CcTIDS9R | tcgcccttgctcaccatggatccCTTCTGCCTCTTGTAAATCTTGCCCA |
| pAN580-CcTIDS10F | ttaagtccggagctagctctagaATGGCTGCCATCCTAAACCATT |
| pAN580-CcTIDS10R | tcgcccttgctcaccatggatccCTGTGCACAGCGAGTGGC |
| qCcTIDS1F | TTGGGAAGACCGCAGGAAAG |
| qCcTIDS1R | AATTCGCTAATGAAATCAGAGGC |
| qCcTIDS2F | GGAAGCCCACCAATCACAAAG |
| qCcTIDS2R | TCGGAGATAGCCCGGAGGAT |
| qCcTIDS3F | GTGAAGGCTCTTTACAACGAACT |
| qCcTIDS3R | CAGCACCTCTTGCATTGCTTTACT |
| qCcTIDS4F | TAGATGATGCGGATACAAGACG |
| qCcTIDS4R | ATTTGCATTGTTTCGCCAGTAAC |
| qCcTIDS5F | AAGGGATACAGAGGACAAAGGA |
| qCcTIDS5R | CGCCTAGAAATCCGAATATCC |
| qCcTIDS8F | GTGTATAGGGCTGCTGTTTCAGG |
| qCcTIDS8R | TGCTCTTTCGCCTCCTTCCT |
| qCcTIDS9F | GCTGGATTTATTCAATGAGGTT |
| qCcTIDS9R | TGTACTGAACAATGCGACGATA |
| qCcTIDS10F | CGGCATGTGGAGCTATTGTAG |
| qCcTIDS10R | CTAAGCCTTTGCACTTCTTCTTCC |

**
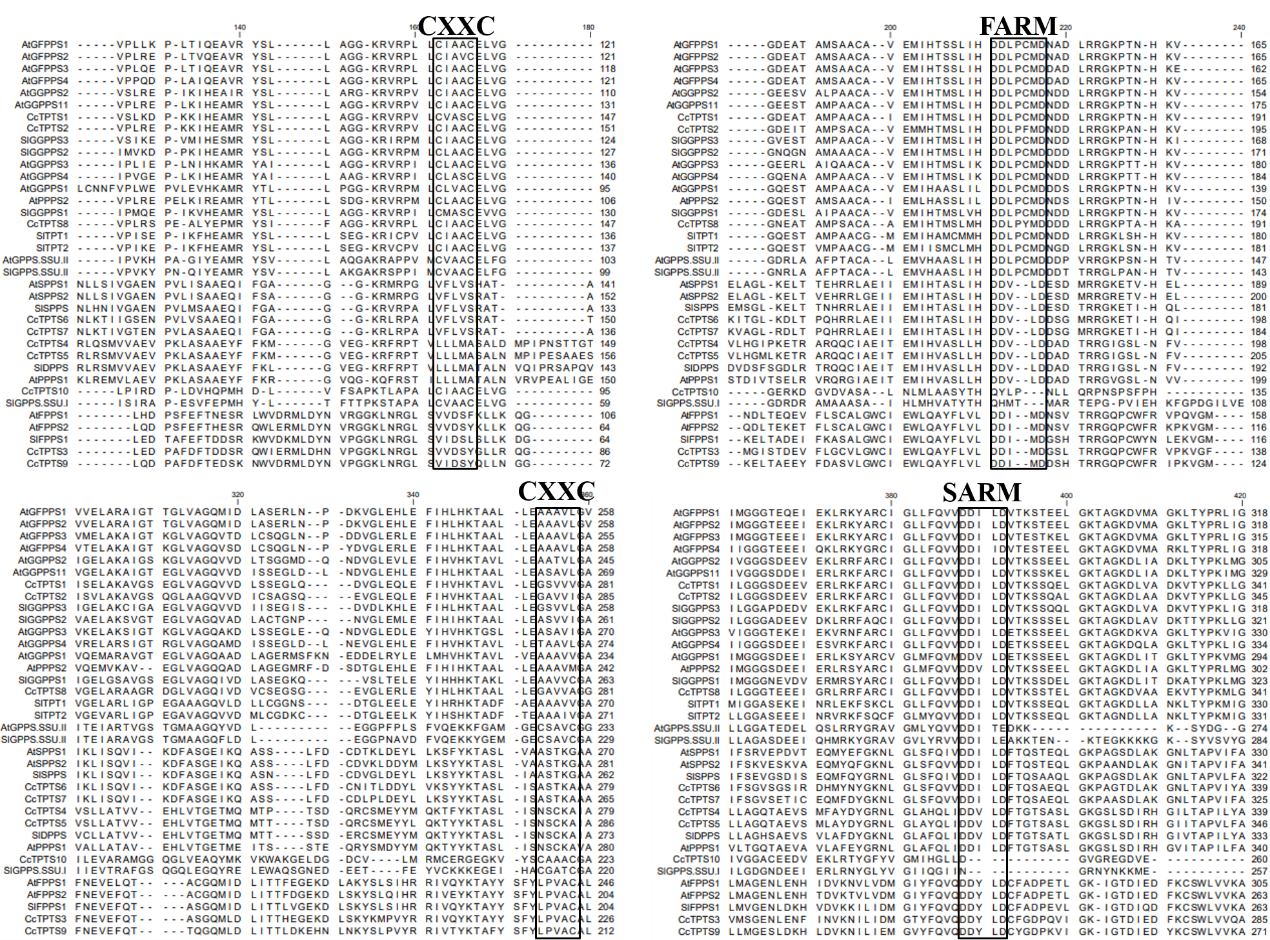
**

**Figure S1** Multi sequence alignment of the CcTIDS amino acid sequence with the AtTIDS and SlTIDS family members. The FARM, SARM and CXXC conserved motifs are marked by a black box.


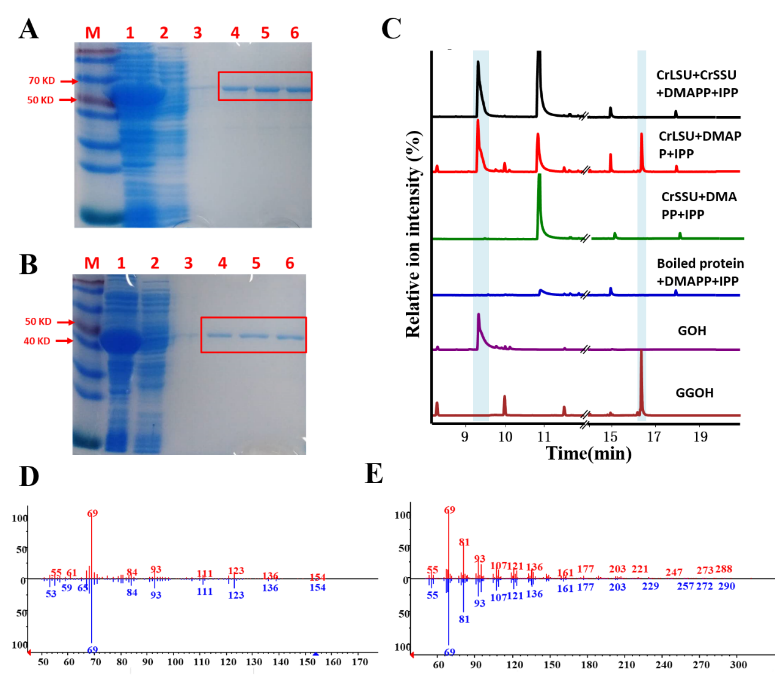


**Figure S2** GC/MS pectrometry profile of CrGPPS.SSU and CrGPPS.LSU in vitro reaction products using IPP and DMAPP as substrates. **A-B** Expression and purification of CrGPPS.LSU (**A**) and CrGPPS.SSU (**B**) recombinant protein from *E. coli* Rosetta (DE3) harboring pET32a(+)-CrGPPS.LSU/CrGPPS.SSU; **1**, total protein after induction; 2, soluble protein; 3-6: purified CrGPPS.LSU or CrGPPS.SSU recombinant protein from the 1st collected tube to the 4th collected

tube; **C** The GC-MS chromatogram of the reaction products generated by CrGPPS.SSU and CrGPPS.LSU protein and the acid hydrolysis products of authentic standards. GOH: Geraniol; GGOH: Geraylgeraniol. **D** Mass spectra of GOH standard (red) and products in theNIST14/Wiley275 library (blue). **E** Mass spectra of GGOH standard (red) and products in theNIST14/Wiley275 library (blue).


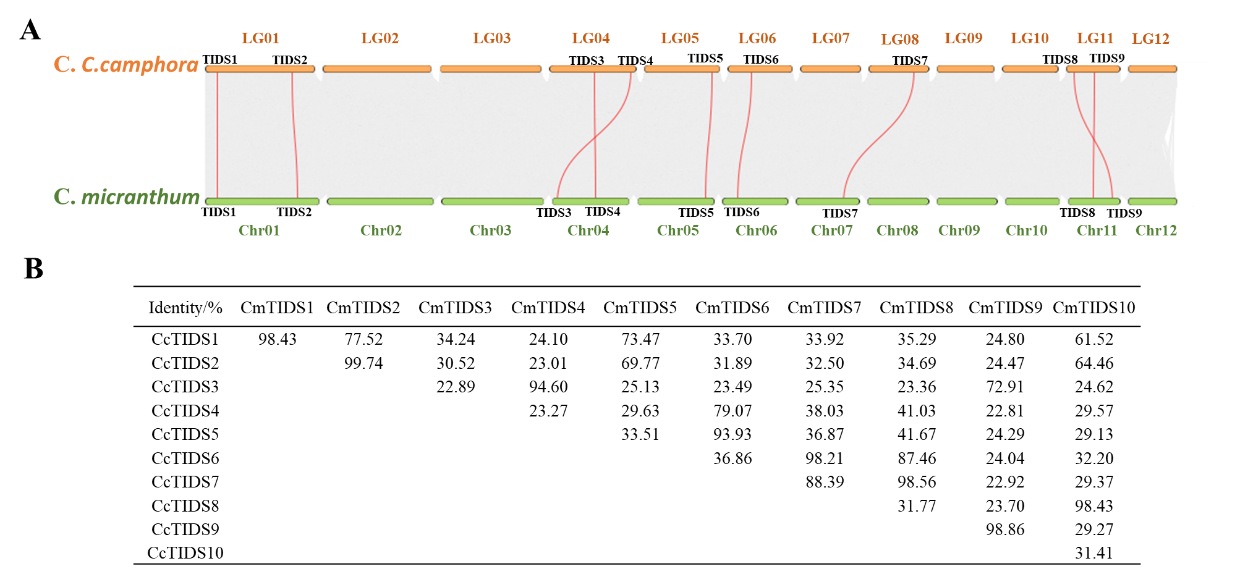


**Figure S3** Collinearity TIDS gene family in *C. camphora* and *C. micranthum* is highlighted with red curved lines over the gray background (genomic collinearity) **(A)**. Identity of TIDS gene family between *C. camphora* and *C. micranthum* **(B)**.
